# Sick leave due to occupational mental disorders in Brazil Northeastern states: an ecological study

**DOI:** 10.47626/1679-4435-2022-1007

**Published:** 2024-08-05

**Authors:** Paula Beatriz de Souza Mendonça, Izabelle Bezerra Costa, Vanessa Cristina de Góes e Silva Faustino da Costa, Janete Lima de Castro

**Affiliations:** 1 Programa de Pós-Graduação em Saúde Coletiva, Universidade Federal do Rio Grande do Norte, Natal, RN, Brazil

**Keywords:** occupational health, mental health, sick leave, saúde do trabalhador, saúde mental, licença médica

## Abstract

**Introduction:**

Although work provides many benefits, occupational mental disorders, such as mental distress, depression, and stress-related illnesses have significantly increased.

**Objectives:**

This study aims to identify and present the spatial distribution of the major mental and behavioral disorders that lead to sick leave in Brazil Northeastern states.

**Methods:**

This descriptive study with an ecological time series design aimed to identify the distribution of occupational mental and behavioral disorders in Brazil Northeastern states. Data collection included downloading information from the Observatório de Segurança e Saúde no Trabalho (SmartLab, Occupational Health and Safety Observatory) from 2012 to 2018. Data were analyzed using Python.

**Results:**

Grants of sick leave according to the type of illness were recorded for nonaccident-related leave (B31) and accident-related leave (B91). Bahia had the highest number of cases reported for B31, as did Rio Grande do Norte for B91. Rio Grande do Norte and Alagoas stood out with the highest rates of sick leave due to mental and behavioral disorders. Phobic-anxiety disorders had the highest number of notifications. The building construction industry had the highest number of work-related notifications.

**Conclusions:**

This study has contributed to identifying the main occupational disorders. Public policies need to be implemented to tackle the public health crisis which directly impacts on domestic social and economic conditions.

## Abstract (pt)

**Introdução:**

Mesmo diante dos benefícios que a atividade laboral proporciona, tem-se observado um aumento significativo no número de trabalhadores com sofrimento psíquico, depressão e doenças associadas ao estresse.

**Objetivos:**

Identificar e apresentar a distribuição espacial por estado do Nordeste dos principais transtornos mentais e comportamentais que geram afastamento da atividade laboral.

**Métodos:**

Realizou-se um estudo descritivo com desenho ecológico de série temporal, cuja finalidade é identificar a distribuição de transtornos mentais e comportamentais em trabalhadores da região Nordeste. Para a coleta de dados, utilizou-se o Observatório de Segurança e Saúde no Trabalho (SmartLab) mediante o *download* das informações notificadas no período de 2012 a 2018. A análise foi realizada por meio da linguagem de programação Python.

**Resultados:**

As concessões de auxílios por afastamento conforme o tipo de doença foram registradas para afastamentos não acidentários (B31) e acidentários (B91). A Bahia apresentou um maior número de casos notificados para B31, assim como o Rio Grande do Norte para B91. O Rio Grande do Norte e Alagoas se destacaram como os estados que concentram o maior percentual de casos de afastamento por transtornos mentais e comportamentais. Os transtornos fóbico-ansiosos obtiveram o maior número de notificações entre os estados. A atividade de construção de edifícios se revelou com os maiores números de notificações relacionadas ao trabalho.

**Conclusões:**

Este estudo contribuiu para a identificação dos principais transtornos que acometem os trabalhadores. É necessário que sejam adotadas políticas públicas no enfrentamento da crise de saúde pública que impacta diretamente as condições sociais e econômicas do país.

## INTRODUCTION

Work makes life meaningful for humans. As an essentially human activity, it is considered a means of subsistence that creates existential perspectives and helps to build and shape the identity and personality of the subject.^1^ This understanding has been the result of an idealization established over time, which is reflected in the daily life of individuals.^2^

The literature on the quality of life of workers points to human work as an important healthpromoting agent for those who perform it. On the other hand, even in the face of favorable conceptions about work and its benefits, the number of workers experiencing psychological distress, depression, and stress-related illnesses has increased significantly - a fact that points to the meaning of work as a source of exhaustion and suffering.^3^

The conditions in workplace, incentives for competition among employees, fewer job vacancies, increased outsourcing of employees, among other factors, have a significant impact on the development of mental and behavioral disorders (MBDs).^4,5^ Mental illness arises as a response of the organism to the external context in which the worker is situated, thus creating a relational existence between work and mental illness.^6^

The International Labor Organization (ILO) and the World Health Organization (WHO) point to data correlating psychosocial factors at work and their effect on the health of workers. According to this study, workers exposed to harmful psychosocial stressors in the workplace tend to have psychiatric and psychosomatic symptoms and changes in subjective well-being and quality of life.^7^

In Brazil, MBDs are seen as a major cause of workrelated sick leave, as they result in disability and low productivity among affected individuals. From this perspective, considering the domestic scenario, in recent years mental illness has ranked third place as a cause of sick leave, becoming a major factor for granting social security benefits.^7^

Brazil Northeastern states rank second in terms of the number of people requesting benefits from the Instituto Nacional do Seguro Social (INSS, Brazil National Social Security Institute). The distribution of benefits granted per zone in 2017 was 20.1% in urban zones and 57.9% in rural areas. As for the distribution of active benefits, 19% went to urban zones and 49.1% to rural areas. Sick leave due to MBDs is categorized in Chapter V of the 10th revision of the International Classification of Diseases and Related Health Problems (ICD-10).^8^

Sick leave due to MBDs not only has an economic impact on society and workers, but also affects their health and quality of life, incapacitating them for work. In this context, the following research question was posed for the study: “What is the spatial distribution and which MBDs most often cause workers to take time off work in the Northeastern states?”

Considering the magnitude of the social problem and its repercussions on the occupational healthdisease process, this study aimed to identify and show the spatial distribution of the main MBDs that cause workers to take time off work per Northeastern state.

## METHODS

This is a descriptive study with an ecological time series design, which aimed to identify the distribution of MBDs among workers in Brazil Northeastern states. The dependent variable was the distribution of MBDs among workers categorized according to the Classificação Brasileira de Ocupações (CBO, Brazilian Classification of Occupations). These data were extracted from the profile of sick leave recorded on the INSS Observatório de Segurança e Saúde no Trabalho (SmartLab, Occupational Health and Safety Observatory)^9^ from 2012 to 2018 as sick leave due to MBDs, according to Chapter V ICD-10.

This study followed the Strengthening the Reporting of Observational Studies in Epidemiology (STROBE)^10^ checklist for observational studies.

Brazil is divided into 5 regions: North, Northeast, South, Southeast, and Midwest. It also has 26 states and a Federal District. The projected population for the study area in 2019 was 210,147,125 inhabitants, occupying 8,510,820.623 km^2^, according to population projections made by the Instituto Brasileiro de Geografia e Estatística (IBGE, Brazilian Institute of Geography and Statistics).^11^

Northeastern states, namely Bahia (BA), Pernambuco (PE), Ceará (CE), Maranhão (MA), Paraíba (PB), Rio Grande do Norte (RN), Piauí (PI), Alagoas (AL), and Sergipe (SE) were chosen for two reasons: firstly, because they are the most populous states in the country. Secondly, because they are the second most populous region, with around 56,560,081 inhabitants.^11^ These factors corroborate a greater appreciation of the diversity between the scenarios analyzed and the factors associated with their occurrence.

We included records of MBDs among workers classified by the CBO who had been absent from work due to MBDs. The information was downloaded from SmartLab^9^ in March 2020. The SmartLab spreadsheets present statistical data on the information presented by SmartLab.

We obtained population data for 2018 for each state from the IBGE.^11^ As a dependent variable, we obtained the general coefficient of MBDs and the variables recorded in SmartLab in the section Perfil dos Afastamentos - INSS (Sick leave profile - INSS), which includes sick leave per injury; leave due to external causes; leave per type of disease; leave due to the international classification of diseases and economic activities. Sick leave was subclassified into accident-related leave (B91) and nonaccident-related leave (B31), according to the SmartLab classification.^9^

This analysis was performed by building an algorithm using Python (version 3.7.6) and Python Matplotlib package for generating graphics (https://matplotlib.org/), Matplotlib Basemap tool for generating maps (https://matplotlib.org/basemap/index.html), and Shapefile gadm36 BRA 1.shp (https://gadm.org/index.html) to generate the state borders.

The following equation was used to calculate the local percentage for each state:


Local rate (P)=number of cases of a specific disorder (n)/number of inhabitants of a given state (N)×100,i.e. P=n/N×100.

The rate for each state was represented by a specific color on a reference color bar (separate color scale for B31 and B91). As the number of inhabitants in each state and, consequently, the number of cases are directly related to its territorial extension, we assessed whether there was a relationship between the number of cases and the number of inhabitants, observing a parameter that could be compared among states.

This study did not require approval from the Research Ethics Committee (REC), as it used public domain data, in accordance with Resolution 510/2016 of the Conselho Nacional de Saúde (National Health Council).

## RESULTS

The group sick leave due to MBDs represented a significant number of 135,800 cases for B31 and 12,800 for B91 among Northeastern states, which were notified between 2012 and 2018.

Among the Northeastern states, BA and RN stand out as having the highest number of cases reported for the variable sick leave, according to ICD-10. BA had the highest number for B31, with 33,158 cases, and RN, for B91, with 2,441 cases.

Comparing the ratio between the number of cases and the number of inhabitants, RN showed the highest rate of MBDs compared to the other states, representing 0.67% for B31 and 0.07% for B91. MA had the lowest notification rate: 0.16% for B31 and 0.008% for B91.

The major illnesses that result in workers taking time off work listed in Chapter V include depressive episode (F32); reaction to severe stress, and adjustment disorders (F43); Alcohol related disorders (F10); bipolar affective disorder (F31); schizophrenia (F20); phobic-anxiety disorders (F40); and Other mental disorders due to brain damage and dysfunction and to physical disease (F06).

**Figure 1:**
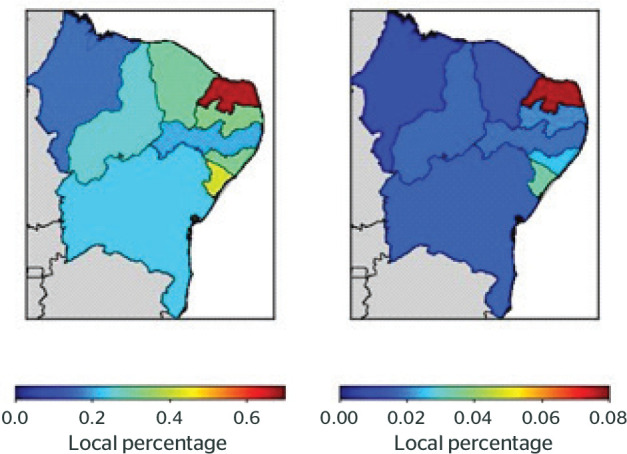
Percentage of reported cases in relation to the number of inhabitants by state, sick leave according to type of illness (Chapter V of the 10th revision of the International Classification of Diseases and Related Health Problems [ICD-10]) between 2012 and 2018. B91 = accident-related sick leave; B31 = nonaccident-related sick leave.

It was observed that the number of sick leave cases compared to the number of inhabitants points to a disparity in a less populous state, with a higher number of sick leave cases for B31 and B91 - compared to larger states, as shown in Figure 1. According to the data analyzed, Figure 1 shows that RN with the highest rate of workers lost due to MBDs among the diseases presented in Chapter V.

**Figure 2:**
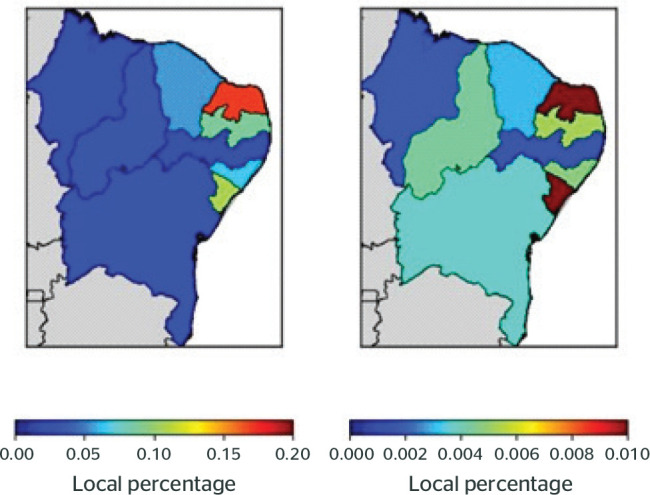
Rate of cases reported in relation to the number of inhabitants by state for phobic-anxious disorders (F40) B91 (accident-related sick leave) and B31 (nonaccident-related sick leave) between 2012 and 2018.

Figure 2 shows that the MBDs with the highest number of notifications per state were phobicanxiety disorders (F40) for B91 and B31. RN had the highest number of sick leave notifications per number of inhabitants for these MBDs, with 0.17% of the notifications for B31, and AL, for B91, with 0.022%. PE had the lowest number of notifications for phobicanxious disorders (F40), with 0.023% for B31 and 0.001% for B91.

The economic activities that most expose workers to sick leave due to MBDs were listed according to the economic areas that had the most sick leave notifications. Table 1 shows the 30 areas that received the most notifications among the states. Of all the economic activities actively legalized in Brazil, those with more than 100 notifications due to sick leave for B31 and B91 were considered for the period analyzed.

**Table 1:** List of the economic activities that had the most notifications in Northeastern states for B91 (accident-related leaves) and B31 (nonaccident-related leaves) between 2012 and 2018

Classification	Activity description
1	Credit card management
2	Public administration in general
3	Health management support activities
4	Activities of social rights defense associations
5	Human health care activities not previously specified
6	Hospital care activities
7	Courier activities
8	Teleservice activities
9	Cash/valuables-in-transit activities
10	Surveillance and private security activities
11	Commercial banks
12	Multiple banks with commercial portfolios
13	Savings banks
14	Retail trade in clothing and accessories
15	Retail trade in hardware, wood, and construction materials
16	Retail trade in general merchandise, predominantly food stuff - hypermarkets and supermarkets
17	Condominiums
18	Garment manufacturing, except underwear
19	Building construction
20	Primary education
21	Raw sugar manufacturing
22	Leather shoes manufacturing
23	Footwear manufacturing from materials not previously specified
24	Footwear manufacturing from synthetic materials
25	Sneakers manufacturing of any material
26	Cleaning of buildings and homes
27	Hiring of temporary labor
28	Restaurants and other food and beverage service businesses
29	Fixed-route intercity, interstate, and international collective passenger road transportation
30	Fixed-route municipal and metropolitan area collective passenger road transportation

**Figure 3:**
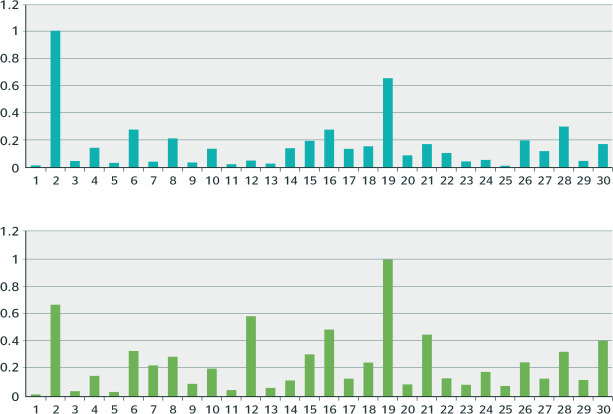
Ratio of economic activities notified by B91 (accident-related leave) and B31 (nonaccident-related leave) between 2012 and 2018. 1 = credit card management; 2 = public administration in general; 3 = health management support activities; 4 = activities of social rights defense associations; 5 = human health care activities not previously specified; 6 = hospital care activities; 7 = courier activities; 8 = teleservice activities; 9 = cash/valuables-in-transit activities; 10 = surveillance and private security activities; 11 = commercial banks; 12 = multiple banks with commercial portfolios; 13 = savings banks; 14 = retail trade in clothing and accessories; 15 = retail trade in hardware, wood, and construction materials; 16 = retail trade in general merchandise, predominantly food stuff - hypermarkets and supermarkets; 17 = condominiums; 18 = garment manufacturing, except underwear; 19 = building construction; 20 = primary education; 21 = raw sugar manufacturing; 22 = leather footwear manufacturing; 23 = footwear manufacturing from materials not previously specified; 24 = footwear manufacturing from synthetic materials; 25 = sneakers manufacturing from any material; 26 = cleaning of buildings and homes; 27 = hiring of temporary labor; 28 = restaurants and other food and beverage service businesses; 29 = fixed-route intercity, interstate, and international collective passenger road transportation; 30 = fixed-route municipal and metropolitan area collective passenger road transportation.

Figure 3 shows the economic activities classified among the 30 main areas with the highest sick leave, organized by dividing the highest rates of each classification between B31 and B91.

Figure 3 shows that public administration in general stands out in the ranking of economic activities, indicating that workers in this activity are more vulnerable to MBDs and B91. The least vulnerable was credit card management, both for B31 and B91. The chart also shows building construction as a sector with greater vulnerability to B91 due to MBDs.

**Figure 4:**
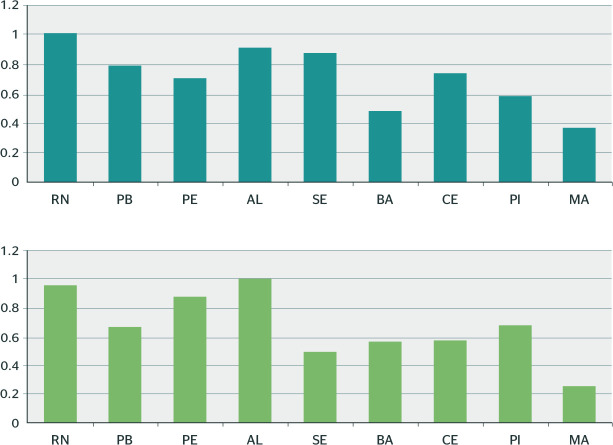
Ratio between states based on B91 (accident-related sick leave) and B31 (nonaccident-related sick leave) in relation to economic activities between 2012 and 2018. AL = Alagoas; BA = Bahia; CE = Ceará; MA = Maranhão; PB = Paraíba; PE = Pernambuco; PI = Piauí; RN = Rio Grande do Norte; SE = Sergipe.

Based on the data for sick leave due to MBDs in each state, the most prevalent economic activities were listed and compared between states. The ratios were then normalized based on the state with the highest rate, as can be seen in Figure 4.

Figure 4 shows that workers from RN suffer more from MBDs for B31 than in other states, and MA had a lower rate of notifications for time off work. The chart also shows that AL had the highest B91 sick leave due to a causal link with exposure at work, and MA had the lowest rate of sick leave.

## DISCUSSION

The number of benefits granted for sick leave due to MBDs in the Northeastern states is a worrying factor and leads to reflection when considering the individuality of the affected worker.^9^ Work can be considered as a health promoter for the worker, reflecting on collective, social, and economic conditions.^12^ Losses due to sick leave have an impact on local, regional, and national economic productivity, directly affecting between 70% and 80% of the economy income from work. The Northeastern states have the second highest number of workers on sick leave.^9,13^

The results show that the states that stood out in terms of sick leave notifications were BA (for B31) and RN (for B91). In a study conducted in BA, there was a growing number of cases of sick leave reported to the INSS, with a higher prevalence of men related to ICD-10 group F40-F48 in 2012. This group was also identified in another study.^12^ In the same study, it was found that the institution that reported the most sick leave was the Centro de Referência Regional em Saúde do Trabalhador (CEREST, Regional Worker’s Health Reference Center), but that it failed to associate the outcome of the causal link with work.^12^ In order to find solutions, the Protocolo de Atenção à Saúde Mental e Trabalho (Care Protocol for Mental Health and Work) was implemented in BA in 2014 to train workers in early diagnosis.^13^

RN has the highest rate of MBDs when compared to the other states. This was also observed in a study that looked at work-related mental disorders (WRMDs). The predominance (36.5%) of notifications between 2007 and 2016 and the concentration of notifications in more populous municipalities such as Natal and Mossoró stand out.^14^ The number of notifications may be linked to these two municipalities having CEREST headquarters and concentrating the main economic activities in the state.^14^

The most prevalent group of MBDs found in this study has also been observed in other studies.^5,13-15^ Among the MBDs that mostly cause occupational sick leave in the Northeastern states, phobic-anxious disorders stand out, with a prevalence for B31 in RN and B91 in AL. An analysis of the distribution of the major MBDs between states also showed that PE had the lowest number of notifications. A study on social security benefits found that the cause of MBDs in AL is linked to the concentration of workers in the state capital, as already observed in RN.^5-15^

The concentration of work in state capitals is a characteristic of the advance of urbanization in Brazil and has been shown in some studies to be a trigger for MBDs.^13-16^ Absenteeism may be related to the demands of the job market and the pace, production, productivity, and quality requirements, the sum of which paralyzes psychic functioning, triggering phobicanxiety disorders.^5^

In terms of economic activity, credit card management was the activity with the least number of workers reporting sick leave for B31 and B91. Public administration was the activity with the most notifications. In a study with civil servants in Tocantins on the correlation between economic activities and MBDs, mental illness accounted for 29.2% of sick leave, highlighting the existence of a larger number, yet not reported due to concerns of exposure to prejudice in the workplace.^16^

A study in BA points out that despite the low number of occupations recorded at the time of notification, public administration and road transportation were the occupations that most affected workers with MBDs between 2007 and 2013.^13^ Some studies have hypothesized that the outcome of illness in public administration is related to the length of service as a civil servant, moving from one state to another in order to take up the position, failure to adapt to the workplace, outsourcing of public sectors, professional frustration, deteriorating working conditions, and being held responsible for service shortcomings.^6,13,16^

The occupation of building construction stood out for B91 and is the second most reported activity for illness in this professional class for B31. Exposure to accidents, depreciation of the workforce and time away from family life may be related to illness. One study pointed to the prevalence of sickness benefit among building construction workers, which showed a link between MBDs and the use of multiple drugs and other psychoactive substances to relieve suffering. It should be noted that the development of compulsions for psychoactive substances is also related to MBDs. Preventive measures should be introduced to deal with the physical and psychological risks associated with this occupation.^17^

As for the ratio between states with work-related sick leave, RN had the highest ratio, followed by AL and SE for nonwork-related sick leave. MA had the best relationship between economic activity and its workers not falling ill due to B31 and B91. As for work-related sick leave and workers mental illness, AL, RN, and PE had the highest rates.

The ratio between RN and AL has already been mentioned. SE and MA stand out, since a study assessing WRMDs between 2007 and 2016 found that SE was the third state with the fewest WRMD cases among the Northeastern states, and MA was the state with the fewest, with 28 cases. PE, on the other hand, was the second state to report the most cases, which is in line with the findings of this study.^14^

Studies that address social and economic variables and the characteristics of the notified workplace are needed, which this study did not intend to do. Discussions on this subject are urgently needed due to the serious global crisis related to the mental illness of the population, as the WHO and the ILO have shown, with the working class being one of the most affected.^16-18^

The limitations of the study include the barriers inherent to ecological studies, such as the impossibility of making causal inferences about exposure to MBDs at an individual level, and not controlling for confounding factors, which can lead to interpretation issues.

## CONCLUSIONS

Understanding the phenomena that cause workers to take time off work is one of the objectives of occupational health policy. This study contributes to the geographical analysis of the Northeastern states in terms of work-related sick leave due to MBDs.

Phobic-anxiety disorders stood out among the MBDs, and RN ranked as having the highest number of notifications among workers for B31 and AL for B91, thus highlighting the need to adopt measures to prevent occupational mental illness and implement programs to promote the psychological health of these individuals.

Public administration represents the highest rate of sick leave in the Northeastern states for B31, which is not correlated with work; however, this may show an underreporting of the causal link with work. Some causes, such as the flexibilization of labor rights, the precariousness of working conditions, and the concentration of employment in state capitals may contribute to increasing sick leave rates due to MBDs in the coming years. The underreporting associated with the lack of preparation for early diagnosis also stand out.

It is essential that the community understands and becomes aware of the health-disease process associated with work. Legislative and executive bodies in the country need to prioritize the introduction of mechanisms related to effective public policies to tackle this serious public health crisis. Mechanisms need to be used to identify the aspects that lead to workers becoming mentally ill and, consequently, taking time off work. The WHO and the ILO highlight the need to identify the main MBDs and economic activities that are leading the working class to become mentally ill, given the urgency of raising discussions around this issue, which has a direct impact on domestic economic and social conditions.

## HOMAGE

The author Vanessa Cristina de Góes e Silva Faustino da Costa participated actively in the development of the article, but unfortunately passed away before its publication. We thank her for her valuable contributions to this study.
